# Predicting Translation Initiation Rates for Designing Synthetic Biology

**DOI:** 10.3389/fbioe.2014.00001

**Published:** 2014-01-20

**Authors:** Benjamin Reeve, Thomas Hargest, Charlie Gilbert, Tom Ellis

**Affiliations:** ^1^Centre for Synthetic Biology and Innovation, Imperial College London, London, UK; ^2^Department of Bioengineering, Imperial College London, London, UK

**Keywords:** synthetic biology, translation rate, translation efficiency, ribosome binding site, 5′-untranslated region, RBS Calculator, RBS Designer, UTR Designer

## Abstract

In synthetic biology, precise control over protein expression is required in order to construct functional biological systems. A core principle of the synthetic biology approach is a model-guided design and based on the biological understanding of the process, models of prokaryotic protein production have been described. Translation initiation rate is a rate-limiting step in protein production from mRNA and is dependent on the sequence of the 5′-untranslated region and the start of the coding sequence. Translation rate calculators are programs that estimate protein translation rates based on the sequence of these regions of an mRNA, and as protein expression is proportional to the rate of translation initiation, such calculators have been shown to give good approximations of protein expression levels. In this review, three currently available translation rate calculators developed for synthetic biology are considered, with limitations and possible future progress discussed.

## Introduction

Synthetic biology is a recently emerged field concerned with engineering complex living systems by assembling individually characterized biological parts in novel combinations. The discipline arose from the discovery of the mathematical logic of gene pairings, as well as from advances made in genetic engineering and recombinant DNA technology (Andrianantoandro et al., 2006). The development of *de novo* DNA synthesis, protein engineering, and the designs of artificial gene networks have greatly contributed to the field’s advancement (Heinemann and Panke, 2006). Synthetic biology seeks to determine the behavior of organisms and their parts, and then to modify and combine them into complete specific tasks. The field is based on the engineering principles of design and fabrication and focuses on the concept of standardized parts (Serrano, 2007). Precise control over the levels of protein expression is an important requirement for the robust operation of complex synthetic circuits built from many parts.

Despite improving characterization and assembly methods, cycles of design, fabrication, and testing in synthetic biology can be slow. Production of circuits with desired properties can require several rounds of testing and modifying, each time editing imperfect parts by mutation or identifying alternatives. Directed evolution has been shown to provide a short cut through this phase (Yokobayashi et al., 2002), but is complicated by the additional work needed to couple networks to selective pressures. Instead, use of predictive mathematical modeling to rationally guide the design of gene networks can greatly improve design cycles to accelerate advances in synthetic biology (Ellis et al., 2009).

Levels of protein expression are affected by both the transcription and translation rates but early genetic engineering approaches usually focused solely on transcription (Lipniacki et al., 2006). The transcription rate’s heavy dependence on the promoter strength and the relative ease of estimating binding affinity of RNA polymerase helped its early popularity (Alper et al., 2005). However, to gain more accurate and efficient control over protein expression translation rates must also be considered.

Translation initiation is one of the major steps in translation and plays a large role in determining the overall translation rate (Laursen et al., 2005; Kudla et al., 2009). While other factors such as the elongation rate and the termination rate also significantly affect translation (Lithwick and Margalit, 2003; Mehra and Hatzimanikatis, 2006), the initiation rate is of particular interest for synthetic biology as it provides a means to tune protein production over many orders of magnitude by only varying the relatively short RNA sequences at the start of mRNAs that determine the initiation rate. Modeling this step is therefore hugely valuable for designing biological systems.

## Ribosome-mRNA Interactions at Initiation

Modeling translation initiation requires an accurate understanding of ribosome interactions with the mRNA 5′-untranslated region (5′-UTR) ahead of protein synthesis. When a ribosome docks with an mRNA to begin translation, only the 30S subunit of the ribosome binds the 5′-UTR. The 16S ribosomal RNA (rRNA) within this subunit binds to a sequence in the 5′-UTR known as the ribosome binding site (RBS), while the initiator transfer RNA (fMET-tRNA) binds to the start codon (AUG) of the protein-coding sequence. The spacing between these sites on the 5′-UTR is important, with a distance of 6–8 nucleotides between the RBS and AUG being optimal (Vellanoweth and Rabinowitz, 1992). Within the RBS, the 3′ end of the 16S rRNA subunit is complementary to a short sequence named the Shine–Dalgarno (SD) sequence.

**Figure 1:**
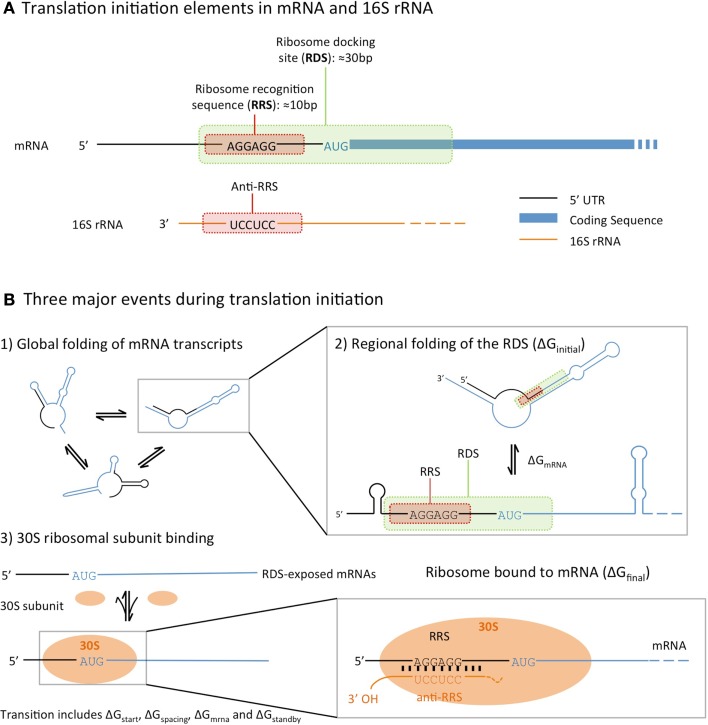
**An illustration of the translational initiation elements encoded in the 5′ untranslated region (5′-UTR) of an mRNA (A), and the three major events that affect prokaryotic translation initiation (B), following the model described by Na and Lee (2010)**. All three calculators estimate translation initiation by considering the difference in free energies between the initial state (unbound mRNA folded into secondary structures) and final (mRNA bound to a ribosome) state.

The factors that influence the rate of translation initiation can be grouped into three categories (Figure 1). Firstly, the global folding and unfolding of transcribed mRNAs, whose secondary structures can hinder the binding of the ribosome: during translation initiation the transcribed mRNA folds in and out of the secondary structures, which may interfere with ribosome binding (de Smit and van Duin, 1990). Secondly, the regional folding and unfolding of nucleotides in the RBS region: the ribosome docking site (RDS), a sequence roughly 30 nucleotides around the start codon, must be unfolded and exposed for the ribosome recognition sequence to bind. Lastly, there is the efficiency of ribosome binding itself, which is determined by the binding affinities between the SD sequence and the complementary 16S rRNA anti-SD sequence (Na et al., 2010).

## Ribosome Binding Models and Calculators

Three different translation rate calculators have been developed. The first, released in 2009 and updated in 2011 is the RBS Calculator (Salis et al., 2009). Next is the RBS Designer (Na and Lee, 2010) and in 2013, Seo et al., developed the UTR Designer. The RBS Calculator uses a statistical thermodynamic model considering free energies for key molecular interactions in translation initiation to give an estimation of translation rate. The UTR Designer uses a very similar model while the RBS Designer makes similar free energy calculations but has a somewhat different method for calculating the translation rate. To find free energy values for mRNA secondary structures and interactions between mRNA and rRNA, all three use secondary software suites. Version 1.0 of the RBS Calculator and the UTR Designer use the NUPACK suite (Zadeh et al., 2011). Version 1.1 of the RBS Calculators instead employs ViennaRNA (Gruber et al., 2008). The RBS Designer uses UNAFold (Markham and Zuker, 2008).

All the translation rate calculators use a proportional scale for their estimated translation initiation rate rather than any definitive units. For example, a predicted output of 500 should produce 10 times more protein than an output of 50, if all other effects are equal. The relative scales are not the same between the different calculators. The three calculators have been initially designed to predict translation initiation rates and estimate protein expression from a given mRNA sequence. This feature is known as “reverse-engineering” as the sequence has been pre-defined and a property of this sequence is calculated. Each calculator also incorporates a “forward-engineering” feature, where a 5′-UTR sequence (if required) and coding sequence are inputted with a desired translation initiation rate. An algorithm is then used to generate a suitable RBS sequence to go between the 5′-UTR sequence and coding sequence to give the desired rate. To accomplish this, a random RBS seed sequence is created and varied until the translation rate matches the desired rate. Each calculator has its own algorithm for efficiently generating and selecting suitable sequences from the combinatorially huge number of possibilities.

### The RBS Calculator

Predicting the rate of translation initiation for different 5′-UTR sequences requires a biophysical model of the process. To do this, Salis et al., developed an equilibrium statistical thermodynamic model using previously characterized free energies of key molecular interactions involved in translation initiation (Salis et al., 2009; Salis, 2011). The model describes two states, an initial state in which a free 30S complex and folded mRNA strand exist and a final state in which the assembled 30S initiation complex is attached to the mRNA. These states are separated by a reversible transition. The two states exhibit a change in the Gibbs free energy, usually labeled as Δ*G*_total_. This is comprised of five different Δ*G* components, each governed by a particular aspect of the binding of the ribosome to the appropriate mRNA sections. The five components are Δ*G*_start_, the energy released when the start codon of the coding sequence hybridizes to the initiator tRNA; Δ*G*_standby_, the work required to unfold secondary structures that sequester a standby site (usually located four nucleotides upstream of the RBS); Δ*G*_spacing_, which depends on the space between the start codon and the SD sequence (preferably five nucleotides); Δ*G*_mrna_, the work required to unfold the local mRNA sequence when it folds to its most stable secondary structure; and Δ*G*_mRNA:rRNA_, the energy released when the SD sequence hybridizes to the 16S rRNA anti-SD. Δ*G*_total_ is related to these Δ*G* terms by the relationship below.

$$\begin{array}{llll} \Delta G_{\mathrm{total}} & =\Delta G_{\mathrm{final}}-\Delta G_{\mathrm{initial}}=\left(\Delta G_{\mathrm{mRNA}:\mathrm{rRNA}}+\Delta G_{\mathrm{start}}\right.\; & & \;\\ & \left.\;+\Delta G_{\mathrm{spacing}}-\Delta G_{\mathrm{standby}}\right)-\Delta G_{\mathrm{mRNA}}\; & & \; \end{array}$$

The translation initiation rate relates to Δ*G*_total_ according to the exponential relationship $r\propto e^{-\beta \Delta G_{\mathrm{total}}},$ where *r* is the translation initiation rate and β is the Boltzmann factor for the system. Similarly, the total protein expression *E* is proportional to the translation initiation rate *r* by a constant *k*, which accounts for ribosomal and mRNA interactions independent of the 5′-UTR sequence and parameters unaffected by translation (Salis, 2011).

The currently available RBS Calculator (Version 1.1) released in 2011, uses the ViennaRNA suite (Gruber et al., 2008) rather than NUPACK (Dirks et al., 2007) for RNA free energy calculations. It also features a modified ribosome footprint length and a more accurate calculation of final state’s free energy by better determination of Δ*G*_mRNA:rRNA_ and Δ*G*_standby_. A further update (Version 2.0) is expected in 2014 based on new research that takes into account the accessible RNA surface at the 5′-UTR (Espah Borujeni et al., 2013).

The Salis Lab RBS Calculator is run from a web-based server and can be found at https://salis.psu.edu/software/. The results page for reverse-engineering shows the entire inputted mRNA sequence, highlighting any possible start codons. For each possible start codon the calculated translation initiation rate is given, followed by the Δ*G*_total_ and all component Δ*G* values. Also, as an advantage over other software, an estimation of confidence is given. A green result indicates relatively high confidence, while various error codes indicate potential inaccuracies. For example, there may be multiple closely spaced or overlapping start codons that could cause unpredictable ribosome–ribosome interactions.

For forward-engineering, the thermodynamic model is combined with a stochastic optimization method to design synthetic sequence. A particular translation rate may be chosen or the “maximize” function selected to give the highest possible translation rate for the given coding sequence. By accurately considering the context effects the software can design synthetic RBSs far stronger than previously possible by manual design or by copying strong natural sequences. A benefit of the forward-engineering mode is the ability to only design synthetic sequences that always satisfy the model’s assumptions, which leads to higher predictive accuracy (Salis, 2011). Constraints may also be placed on the required sequence during forward-engineering. The 5′-UTR can be entered with specification of which nucleotides may be altered according to the UIPAC degenerate nucleotide code. For example, when an *Xba*I restriction site must be located near the start codon, the sequence NNTCTAGANNNNNNN could be inputted (Salis, 2011). The RBS Calculator can also undertake the computationally intensive tasks of specifying and evaluating RBS libraries. Outputs may be degenerate sequences with possible translation rates over a specified range or the possible output range of a chosen degenerate sequence can be calculated.

### The UTR Designer

Seo et al. (2013) developed the UTR Designer following their previous research findings on the importance of 5′-UTR sequences (Park et al., 2007; Seo et al., 2009). The UTR Designer uses a model quite similar to Salis et al., and also defines five Δ*G* terms. The UTR Designer uses a Δ*G*_spacing_ and Δ*G*_start_ term, and utilizes a Δ*G*_SD_ term in lieu of Salis’ Δ*G*_mRNA:rRNA_. Rather than Δ*G*_standby_ and Δ*G*_mRNA_, the UTR Designer uses terms called Δ*G*_direct_ and Δ*G*_indirect_. The former represents the energy released when the 30S subunit directly binds the mRNA when the translation initiation region exists in a transiently unfolded state. The latter represents the energy released when the 30S subunit non-specifically binds and slides into the translation initiation region as it unfolds. The two situations that result in Δ*G*_direct_ and Δ*G*_indirect_ cannot both occur simultaneously. As a result, a population vector α is used to indicate the likelihood of either occurring. The total difference in Δ*G*, denoted Δ*G*_total_ in the RBS Calculator, is called Δ*G*_UTR_ for the UTR Designer, and is defined as a Δ*G*_final_ − Δ*G*_intial_. Δ*G*_initial_ is defined as αΔ*G*_direct_ + (1 − α) Δ*G*_indirect_ where α is the previously described population vector experimentally determined to be ~0.5. Δ*G*_final_ is defined as Δ*G*_start_ + Δ*G*_SD_ + Δ*G*_spacing_.

The output Δ*G*_UTR_ term, equal to Δ*G*_final_ − Δ*G*_intial_, is used to estimate relative translation rate (*r*) using the Salis et al. (2009) exponential relationship $r\propto e^{-\beta \Delta G_{\mathrm{UTR}}}$.

The UTR Designer can be found at http://sbi.postech.ac.kr/rbs. The reverse-engineering results page gives the imputed sequences with position of each possible start codon and the predicted core RBS sequence highlighted. The calculator indicates the standby location where the ribosome may bind to contribute to the Δ*G*_indirect_ term and the nucleotide spacing between the start codon and the RBS. The forward-engineering mode designs an optimal sequence to achieve a given expression level. Unlike other calculators, however, the UTR Designer can also alter the codons of the coding sequence in order to reduce secondary structures and improve translation rate when the variations in 5’-UTR cannot satisfy the desired expression levels. It also features a UTR Library Designer that designs degenerate sequences to give translation rates across a specified range.

### The RBS Designer

A third translation rate calculator, the RBS Designer, was developed by Na and Lee (2010). The model is somewhat different to the others described, using “translation efficiency” to predict protein expression. This is the probability that a given mRNA is bound to a free ribosome. The model defines a Ribosome Recognizing Sequence (RRS) as a 10 nucleotide sequence that includes the SD sequence and is the reverse complement of the 3′ end of the 16S rRNA (anti-RRS). It also defines a RDS of 30 nucleotides that surrounds the start codon where the ribosome physically connects to the mRNA. The model first determines which mRNA sequence is the RRS by determining the minimum hybridization energy to the ribosome’s anti-RRS sequence using UNAFold (Markham and Zuker, 2008). This also gives a ribosome binding affinity value for that particular RRS.

Possible mRNA secondary structures are next considered and the Δ*G* of each are determined using UNAFold. For each structure, an RDS exposure probability (the probability that the RDS will be accessible to the ribosome) is determined by calculating individual nucleotide unpairing probabilities for each nucleotide within the RDS. Individual probabilities of each structure forming are calculated then multiplied by each structure’s individual RDS exposure probability. All terms are then summed to give the total exposure probability for the RDS.

Ribosome binding is modeled with ordinary differential equations and the steady state is assumed. The probability of a given mRNA being bound to a ribosome (translation efficiency) is then calculated from the total RDS exposure probability and ribosome binding affinity, with other parameters taken from the literature. This translation efficiency is approximately proportional to protein production level (de Smit and van Duin, 1990).

The RBS Designer must be downloaded and run locally. Installation instructions and relevant links can be found at http://rbs.kaist.ac.kr/. A notable difference compared to other software is the requirement for at least 300 nucleotides of mRNA sequence. This allows better prediction of secondary structures by considering long-range interaction but is more computationally intensive. The RBS Designer can estimate translation rate for a given mRNA sequence in reverse-engineering mode and in forward-engineering mode it uses a genetic algorithm to vary and select optimal nucleotide sequence, designing a 5′-UTR sequence to give a specified translation rate. Unlike the other calculators, however, the program lacks any library design features.

## Discussion

Each of the currently available calculators show similarly accurate predictions compared with experimental data in their respective publications. The RBS Calculator was tested with 29 synthetic RBSs and predictions correlated well with experimental results with *R*^2^ = 0.84 (Salis, 2011). The UTR Designer was tested with 69 different mRNAs including four different coding sequences and gave *R*^2^ = 0.81 (Seo et al., 2013). The RBS Designer was only tested with 22 designed sequences but data correlated very well with *R*^2^ = 0.87 (Na and Lee, 2010). With these high levels of accuracy the software can be hugely valuable to synthetic biologists for informing and checking designs and for creating new designs with predictable outputs.

There are several areas of improvement for RBS Calculators. Salis acknowledges several limitations of his model and these simplifications are also present in the other models (Salis, 2011). The models do not accurately account for the interaction between the mRNA and ribosomal S1 protein. This protein helps destabilize mRNA secondary structures and is crucial for translation when SD sequences are weak. Its mechanism of function is, however, poorly understood (Qu et al., 2012). Other biological phenomena not included are the effects of antisense RNA or RNAse binding sites, and translational coupling between multiple coding sequences in an operon, where translation of neighboring genes are dependent on each other, such as when an RBS and upstream coding sequence overlap. All the current models make the simple assumption that all start codons are independently translated, ignoring the potential for coupling or interference between closely spaced start codons.

Accounting for these limitations and refining the parameters of the models will lead to improvements in accuracy. There is also room for widening applicability. All calculators were designed for use with *E. coli* and acknowledge that they would not be as accurate for other organisms (though models should hold for similar Gram-negative bacteria). With further testing, models could be adapted to include Gram-positive bacteria. These cells exhibit differences in translational machinery with a major difference in optimum spacing requirements between the SD sequence and the start codon, which would significantly affect the Δ*G*_spacing_ terms (Vellanoweth and Rabinowitz, 1992). Many environmental factors could also be considered. Current calculators only consider interactions at 37°C whereas many well-researched organisms are cultured at different temperatures such as bacilli grown at 30°C or industrially useful thermophiles at 60°C or higher. Temperature changes, at least theoretically, would have a significant effect on RNA folding and thus translation initiation rate. Likewise, RNA folding characteristics are also presumably affected significantly by changes such as the salt concentrations of the cytosol and the molecular crowding within cells of different sizes.

## Conclusion

Ribosome binding site calculators are increasingly valuable tools for synthetic biologists. They allow translation strengths to be estimated from the mRNA sequence so genetic designs can be better informed. Three calculators have been created with two (RBS Calculator and UTR Designer) using a thermodynamic model and run from online servers, and a third (RBS Designer) using a steady-state kinetic model with a downloadable application (Table 1). All of the models seek to simplify the complex natural phenomenon of translation and will continue to be improved and refined to increase predictive accuracy.

**Table 1:** **Key differences between the calculators**.

Feature	RBS Calculator (Salis et al., 2009; Salis, 2011)	UTR Designer (Seo et al., 2013)	RBS Designer (Na and Lee, 2010)
Location	Online	Online	Locally run
Forward and reverse engineering	Yes	Yes	Yes
RBS library design	Yes	Yes	No
External software used for RNA free energy calculations	ViennaRNA (v1.1)	NUPACK	UNAfold
	NUPACK (v1.0)	
Unique selling points	The most frequently updated model, gives indications of confidence	Can edit codon usage to limit unwanted secondary structures	Considers very long-range interactions within the mRNA

## Conflict of Interest Statement

The authors declare that the research was conducted in the absence of any commercial or financial relationships that could be construed as a potential conflict of interest.
